# Assessing Gingival Smile and Muscle Strength

**DOI:** 10.1590/0103-644020266640

**Published:** 2026-08-24

**Authors:** Karinne Bueno Antunes, Luiz Eduardo Monteiro Dias da Rocha, Ramon Silva dos Santos, Lucio Souza Gonçalves, Ricardo Guimarães Fischer

**Affiliations:** Department of Periodontology, Dental School Rio de Janeiro State University, Rio de Janeiro, Brazil; 2 Department of Periodontology. Dental School Rio de Janeiro State University, Rio de Janeiro, Brazil; 3 Laboratory of Electronic Instrumentation and Analytical Techniques, Armando Dias Tavares Institute of Physics, Rio de Janeiro State University, Rio de Janeiro, Brazil; 4 Professor of Periodontology, Estácio de Sá University, Rio de Janeiro, Brazil; 5 Professor of Periodontology, Dental School Rio de Janeiro State University. Rio de Janeiro, Brazil

**Keywords:** muscle strength, upper lip, hyperactivity, gummy smile

## Abstract

A gummy smile (GS) may result from dental, skeletal, or muscular etiologies, with lip hyperactivity (LH) being the most prevalent. Currently, lip muscle strength assessment relies on empirical methods, compromising the precision of certain treatments. This study aimed to evaluate muscle strength related to smiling, establish correlations between lip movement and both individual and total muscle force, and determine the prevalence of GS etiologies. A device was developed to measure the strength of each lip muscle during smiling. A total of 89 individuals (mean age 28 ± 8.4 years; 80.9% female) were divided into four groups: G1 - Upper lip hyperactivity (9%); G2 - Dental/skeletal causes (19.1%); G3 - Control (33.7%); G4 - Hyperactivity combined with other etiologies (38.2%). Statistical analysis included ANOVA, Tukey’s post hoc, and chi-square tests. LH, alone or combined, was the most prevalent etiology (68.2%) and presented 30% greater muscle strength than other groups. G4 exhibited significantly higher strength across all six muscles evaluated compared to G2 and G3. The zygomaticus major muscle in G1 was significantly stronger than in G2 and G3. Lip translation exhibited significant differences in force based on displacement (p <0.001), with the most substantial force observed from 8 mm of displacement. LH was identified as the primary etiology of GS, with individuals displaying LH showing greater muscle strength compared to other etiologies or controls.

## Resumo

Um sorriso gengival (SG) pode resultar de etiologias dentárias, esqueléticas ou musculares, sendo a hiperatividade labial (HL) a mais prevalente. Atualmente, a avaliação da força muscular labial é realizada de forma empírica, comprometendo a precisão de determinados tratamentos. Este estudo objetivou avaliar a força muscular relacionada ao ato de sorrir, estabelecer correlações entre o movimento labial e as forças musculares individual e total, bem como determinar a prevalência das etiologias do SG. Um dispositivo foi desenvolvido para medir a força de cada músculo labial durante o sorriso. O estudo incluiu 89 indivíduos (idade média de 28 ± 8,4 anos; 80,9% do sexo feminino), divididos em quatro grupos. A HL, isolada ou combinada, foi a etiologia mais prevalente (68,2%), apresentando força muscular 30% superior aos demais grupos. Houve correlação significativa entre deslocamento labial, exposição gengival e força muscular

**Figure:**
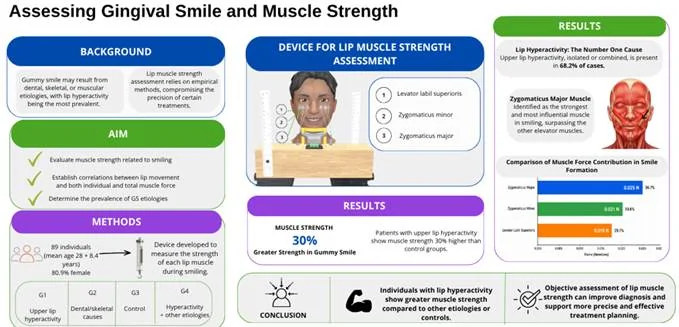

## Introduction

Excessive gingival exposure (GE), defined as more than 2.0 mm of visible gingiva during smiling, ^1^, significantly impacts aesthetics and self-esteem, especially in a society driven by beauty standards, ^2^. This is particularly poignant in our society, where media perpetuates predefined standards of beauty and happiness and where aesthetics can influence personal, social, and professional relationships ^2^.

This condition affects approximately 10% of individuals aged 20-30 years, predominantly women ^3^. GS etiologies can be dentoalveolar or non-dentoalveolar ^4^, with the latter often related to hyperactive lips, short upper lips, or vertical maxillary excess ^4^. This gender disparity in prevalence not only highlights the unique challenges faced by women but also calls for our empathy and consideration in this context ^3^.

The complexity of excessive GE, with its various etiologies that can be single or multifactorial, underscores the importance of a precise diagnosis for effective, individualized treatment. It is common for a patient to require a combination of treatments, highlighting the need for a comprehensive understanding of this condition ^4^.

The upper lip’s position and movement significantly influence gingival display, with hyperactive lips translating 1.5 to 2 times more than normal, whose normal measurement varies from 6 to 8 mm from the resting position to the wide smile ^5^ ^,^ ^6^. This is one of the leading causes of gummy smiles ^7^.

The high prevalence of LH in patients with excessive GE underscores the potential benefits of treatment modalities to limit upper lip movement ^7^ ^,^ ^8^. Surgical lip repositioning, a widely used procedure ^9^ ^,^ ^10^, can lead to recurrence ^4^. Less invasive non-surgical techniques, such as botulinum toxin type A in the upper lip muscles, offer efficacious and minimally invasive temporary therapy with a short-term effect and stability of 2 to 3 months ^11^ ^,^ ^12^ ^,^ ^13^. The association of toxins in pre-surgery has shown better results in terms of stability ^14^.

Although gingival smiles have been studied over the last few years, when the cause is hyperactivity of the upper lip, few studies with effective correction techniques allow for stable results. As a result, establishing a well-defined protocol is very important, mainly focusing on the individualized muscle activity and strength of each patient involved in this process of upper lip displacement when smiling. To measure the strength of the muscles involved in smiling and understand their interrelationships and importance in defining treatment, additional research in the literature is essential. Therefore, this study aimed to objectively measure the strength of each muscle involved in smiling, establish correlations with lip displacement and gingival exposure, and determine the prevalence of GS etiologies.

## Methods

This cross-sectional study included 89 individuals divided into four groups. Group 1 (G1) consisted of individuals with gingival smiles with isolated upper LH etiology; Group 2 (G2) individuals with gingival smiles with other causes, dental or skeletal; Group 3 (G3) individuals without gingival smiles (control group), Group 4 (G4) individuals with LH combined with one or more etiology. Clinical examinations were performed at the Dental School, Rio de Janeiro State University (UERJ), Brazil. A convenience sample was used, due to the absence of previous studies measuring lip muscle strength with similar methodology, an a priori sample size calculation was not feasible. 

### Diagnosis 

**Figure 1:**
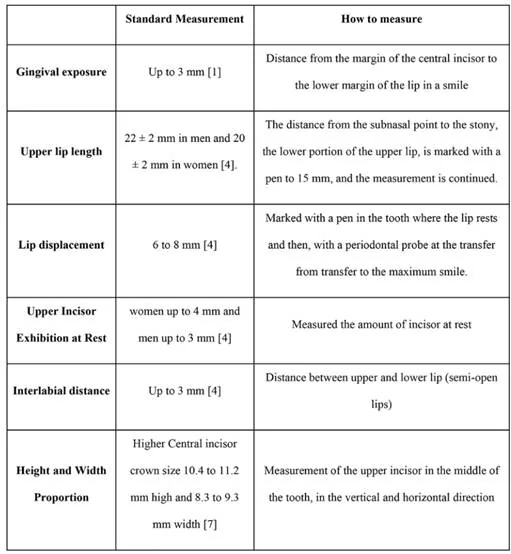
Clinical parameters for the evaluation of the gummy smile used in the study.

Evaluation included clinical measurements of central incisor crown length, lip displacement, lip height, GE, upper lip and facial symmetry during smiling, teeth exposure at rest, lip seal at rest, and increased interlabial distance, using a calibrated periodontal probe (Hu-Friedy®, Chicago, USA) and operator was calibrated with the parameters (Figure 1). 

### Sample selection

Patients were recruited through internal advertising at the Dental School (UERJ), social media, and the University's YouTube channel. For the test groups, 59 patients diagnosed with a gummy smile were randomly selected according to demand. The control group included 30 patients without gummy smiles. The Ethics and Research Committee of the Pedro Ernesto University Hospital / UERJ approved the study under number 5.091.120.

### Inclusion/Exclusion Criteria 

The inclusion criteria for the test group were age between 18 and 59 and GE ≥3 mm. The patients were divided into subgroups based on the type of etiology. The control group had GE <3 mm. Exclusion criteria were individuals with a history of neurological disease, use of muscle relaxants, and those who had received botulinum toxin injections within the previous 12 months.

### Measurement equipment 

In partnership with the Electronic Instrumentation and Analytical Techniques Laboratory at the Armando Dias Tavares Physics Institute (UERJ), a mechanical prototype was developed to measure the lip muscle force when smiling. The methodology consisted of a prototype, a camera to capture the lip displacement while smiling on video, and software to analyze the spring's displacement.

**Figure 2:**
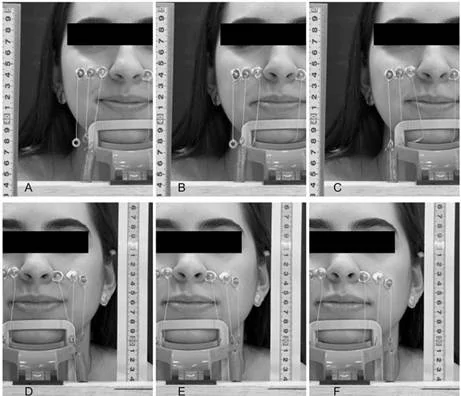
Demonstration of equipment being used in a participant of the study, right (R) and left (L) sides: A) upper lip elevator - R; B) minor zygomatic - R; C) major zygomatic - R; D) lifter of the upper lip - L; E) minor zygomatic - L; F) major zygomatic - L.

This study analyzed 3 muscles on each side of the face (right and left), which participate in the stages of smile formation (Figure 2). The 6 muscles analyzed in this study were: a) upper right lip elevator (muscle 1); b) right minor zygomatic (muscle 2); c) left zygomatic major (muscle 6); d) upper left lip elevator (muscle 4); e) left minor zygomatic (muscle 5) and left zygomatic major (muscle 6).

### Muscle force calculation

The force exerted by the muscle when the individual smiles was calculated using the physical principle of Hooke's law ^15^. Through an experimental setup using a spring, the relationship between the force exerted by the muscle when smiling and the restoring force of this spring can be obtained. After recording the entire experimental process, the videos were analyzed using Tracker® software. By differentiating between the position of the spring when smiling maximally and when the face is relaxed, it is possible to check the spring's displacement caused by the muscle's force when smiling. The average displacement of the spring for each of these muscles was measured, and then the muscle force was calculated using the formula |𝐹| = 𝑘. 𝑦, where the muscle force multiplies the spring displacement (y) with the spring's elastic constant (k). 

### Statistical analysis

The SPSS^®^ 28.0 software was used to analyze the data. The normality of the quantitative variables was checked using the Shapiro-Wilk tests and graphical analysis. The mean forces of the muscles in four groups were compared using analysis of variance (ANOVA), followed by Tukey's post hoc test. The qualitative variables were compared using the chi-square test. The level of statistical significance established was 5% (p <0.05).

## Results

The sample (n=89) had a mean age of 28 ± 8.4 years old, 72 females (80.9%) and 17 males (19.1%). This ratio was 85% and 15% in the gummy smile groups. The 19-30 age group accounted for 78.7% of the sample. Individuals in G1 were significantly older than the other groups. The mean age and standard deviation with the number (n) of patients were G1(8), 37±15; G2 (17), 25±4; G3 (30), 25±6; G4 (34), 28±8.

Table 1 describes the prevalence of different etiologies of gummy smiles. LH alone or in combination was found in 68.2% of the 59 patients with gummy smiles. One individual with vertical maxillary excess and LH had the most considerable GE in the sample, 10 mm. LD had an overall average of 8.6 mm (±1.9). The average for all individuals with LH was 10.2 mm (±1.5), close to G1 and G4. G1 presented significantly higher LD than groups G2 and G3 (p <0.001). The same was observed for G4 when compared to G2 and G3 (p <0.001) (Table 2). The total sample's mean GE was 3 mm (±2.4). G1, G2 and G4 had significantly higher GE than G3 (p <0.001). G4 also had a significantly higher GE than G2 and G3 (p <0.001)

**Table 1:** Prevalence of the different etiologies of gingival smiles isolated and combined (n = 59).

Etiologies	n (%)
Lip hyperactivity	8 (13.5)
Altered passive eruption	11 (18.6)
Vertical maxillary excess	4 (6.8)
Altered passive eruption+ Lip hyperactivity	12 (20.3)
Altered passive eruption+ Vertical maxillary excess	4 (6.8)
Lip hyperactivity +Vertical maxillary excess	14 (23.7)
Three etiologies combined	6 (10.2)

**Table 2:** Mean (± SD) lip displacement (LD), gingival exposure (GE) and upper lip length in mm in the different groups.

G	LD	GE	Upper lip length
G1	10.1 ±1.4*	4.1 ± 1.4 ^■^	23.6 ± 2.4
G2	7.8 ± 0.7	3.1± 0,5 ^■^	22.7 ± 3
G3	6.9 ± 0.8	0.2 ± 0.6	22 ± 2.7
G4	10.2 ± 1.6**	5 ± 1.6^■^	23.6 ± 2.6

Note: *significant difference between G1 and G2 and G3 (p <0.001).

** Significant difference between G4 and G2 and G3 (p <0.001).

■ Significant difference between G1, G2 and G4 and G3 (p <0.001).

ANOVA test with Tukey's correction.

The average total force (F) of each individual in the sample was 0.13 ± 0.03 N during the smiling movement. Values were obtained for each muscle on each side (right and left), and G1 and G4 showed the greatest strength. The strengths of the muscles assessed were compared within the different groups. G4 showed significantly higher strengths in the 6 muscles evaluated than G2 and G3 (p <0.001). The major zygomatic muscle on the right and left sides of G1 was significantly more potent than that observed in G2 and G3. In the minor zygomatic muscle on the right side, G1 had a significantly higher F than G2 (p <0.05); on the left side, G1 had a significantly higher F than G2 and G3. There was a tendency for G1's F to be higher than G3's in the upper lip elevator muscle on the right, while on the left, G1's F was significantly higher than G2 and G3's (Table 3).

**Table 3:** Mean (± SD) of each muscle strength (N) and the mean (± SD) of the sum of the strength of the 2 sides in each group (G).

		FLD	FzmD	FZD	FLE	FzmE	FZE	F D+E
G1(n)	8	0.019±0.003	0.021±0.003^*^	0.026±0.004^■^	0.027±0.006^■^	0.028±0.005^■^	0.036±0.008^■^	0.16±0.02^■^
G2 (n)	17	0.016±0.005	0.015±0.005	0.019±0.005	0.018±0.005	0.021±0.007	0.023±0.007	0.11±0.03
G3 (n)	30	0.014±0.003	0.016±0.003	0.019±0.004	0.017±0.006	0.018±0.006	0.021±0.007	0.10±0.02
G4 (n)	34	0.019±0.005^**^	0.023±0.007^**^	0.026±0.005^**^	0.024±0.007^**^	0.027±0.007^**^	0.032±0.008^**^	0.15±0.03^**^

Legend: FLD and FLE - Strength of the upper lip elevator on the right and left sides, respectively;

FzmD and FzmE - Strength of the minor zygomatic on the right and left sides, respectively;

FZD and FZE - Strength of the major zygomatic on the right and left sides respectively;

F D+E - Total strength of the 6 muscles.

Note:* significant difference between G1 and G3 (p <0.05, F1D )

** significant difference between G4 and G2 and G3 (p <0.05 in F1D, F1E, F2E) (p <0.001 in F2D, F3D, F3E, FD+E); ■ significant difference between G 1 and G2 and G3 (p <0.05 in F3D, F1E, F2E, F3E, FD+E). ANOVA test with Tukey's correction.

LD was evaluated using its mean force (Table 4), and the different displacements and forces were compared. A statistically significant difference (p <0.001) was observed between 6 mm and 7 mm, 7 mm and 8 mm, 9 mm and 10 mm, and 9 mm and 11 mm. No significant difference was observed between 8 mm and 9 mm, 10 mm and 11 mm, and 11 mm and 12 mm (Table 4).

The results shown in Table 5 consider the mean (± SD) force for each muscle and the sum of the right and left sides. The strongest muscle was the major zygomatic muscle, regardless of the side of the face and the group. There was a statistical difference between the force of the major zygomatic muscle (FZDE) and the other muscles.

**Table 4:** Mean total force (±SD) in Newtons (N) for each lip displacement (LD) in mm and the corresponding number (n)

LD	6 mm	7 mm	8 mm	9 mm	10 mm	11 mm	12 mm
Mean total force	0.096±0.02*	0.104±0.02*	0.12±0.02	0.14±0.02*	0.16±0.03	0.18±0.02	0.18±0.02
n	12	13	21	14	12	8	8

Note: *significant difference between LD 6 mm and 7 mm (p <0.001);

*between 7 mm and 8 mm (p <0.001);

*between 9 mm and 10 mm (p <0.001).

ANOVA test with Tukey's correction.

**Table 5:** Mean total force (right + left sides) of each muscle involved in smiling (N) in the total sample.

Muscle	Mean Force	SD	Contribution to total force (%)
FLDE	0.019 N	0.006	29.7
FzmDE	0.021 N	0.006	33.6
FZDE	0.025 N	0.007	36.7

FLDE - Combined force of the upper lip elevator (right + left sides).

FzmDE - Combined force of the minor zygomatic muscles.

FZDE - Combined force of the major zygomatic muscles.

The major zygomatic muscle presented significantly higher force compared with the other muscles (**p < 0.001**).

ANOVA test with Tukey's correction.

The average total force of all muscles (right and left) is 0.11 ± 0.02 N at G3 and 0.15 ± 0.03 N for all individuals with LH (G1 and G4). Individuals with LH have an average total force of 0.15 ± 0.03N, which represents a significantly higher force than individuals without LH, such as G3, with a force of 0.11 ± 0.02 N (p <0.001). Pearson's correlation between LD and GE showed a high, statistically significant correlation coefficient (r = 0.78, p <0.001). The total force of the 3 muscles and LD showed a statistically significant correlation coefficient (r = 0.79, p <0.001). The correlation between total force and GE had a moderate and significant correlation coefficient (r=0.57, p <0.001).

## Discussion

This study aimed to evaluate the muscle strength related to the act of smiling, establish a correlation between lip movement and the individual/total force exerted and determine the prevalence of gingival smile etiologies. Our results showed that the major zygomatic is the strongest muscle in gummy smiles, with 36.7% of the total F, followed by the minor zygomatic (33.6%) and the upper lip elevator muscle 29.7%. Moreover, a statistically significant correlation existed between LD, GE, and the total F of the major and minor zygomatic and upper lip elevator muscles. 

Our findings differ from what has been described in the literature, which states that LH is directly associated with muscle hypertonicity, mainly related to the upper lip elevator muscle ^6^ ^,^ ^15^. However, there is subjectivity in assessing the muscles involved in the smile's movement since no study evaluated the muscle's strength. In the present study, we measured the F of each muscle involved in the gingival smile and verified which muscles were the most important. Equipment (patent number BR 10 2023 019317 0) was designed in collaboration with the Electronic Instrumentation and Analytical Techniques Laboratory of the Armando Dias Tavares Physics Institute (UERJ). This prototype was specifically developed for this study with the aim of measuring the strength of each muscle involved in the displacement of the upper lip, enabling the applicability of a classification based on the correlation between muscle strength and LD.

Based on these measurements, it was found that the greater the LD, the greater the total muscle strength (r = 0.79, p <0.001). Although this strength has not previously been measured, studies have reported that the upper lip elevator is the most active muscle, with a 20% increased capacity in patients with gummy smiles (6, 16). The results showed that the mean F of the major zygomatic was 0.025 ± 0.008 N, while the minor zygomatic and upper lip elevator was 0.021 ± 0.007 N and 0.019 ± 0.006 N, respectively. The strength of the major zygomatic was significantly higher (p <0.001) than the minor zygomatic and upper lip elevator muscles.

LH was the most prevalent etiology (68.2%), followed by altered passive eruption (56%) and vertical maxillary excess (47%) among individuals with gummy smiles in this sample. Regarding the classification of LH, it is noted that a shift of approximately 6 to 8 mm from the resting position to a wide smile is considered regular, whereas in hyperactive upper lip, this distance can be 1.5 to 2 times greater, i.e., from 9 mm ^5^. The results indicated that the most significant F started at 8 mm in LD. Based on the observations of the present study, a different classification can be proposed, separating patients with LH into 2 groups. In grade 1, LD ranges between 8 and 9 mm, while in grade 2, LD would be ≥10 mm.

is the primary etiology of the gummy smile.

Gingival smiles are prevalent in 10% of the population, predominating females and negatively impacting self-esteem and quality of life ^17^. Upper LH is the most prevalent etiology, including altered passive eruption, vertical maxillary excess, short lip, and anterior dentoalveolar extrusion (7, 8). In this study, the highest prevalence, isolated or combined among the etiologies above, was LH (68.2%), like other studies ^7^ ^,^ ^8^.

It was observed that many subjects had asymmetrical smiles, 22.5% in the sample. For this reason, the experiment was carried out individually on the left and right sides, as both the face and the smile may not be symmetrical. Further studies will be needed to verify what difference, in mm, between the sides should be considered asymmetry and the relationship with other aspects, such as the dominant side. Then, it would be possible to evaluate different treatments for patients with asymmetrical smiles. The higher prevalence of gummy smiles among women shows sexual dimorphism in this study; 85% were women, while only 15% were men. Other studies reported that the proportion of women with high smile lines was double that of menIn the present study, women outnumbered men by approximately 5.7 to 1 ^9^.

The degree of exposure and age also influence the diagnosis ^18^ since older individuals have reduced muscle tone and consequently reveal their teeth less ^19^. This is why gummy smiles are prevalent in the 20 to 30 age group. However, the present study found that the group with LH also had a higher force and was composed of individuals with a mean age of 37 years, while the control group was younger than the individuals with gummy smiles.

Botulinum toxin is a conservative and immediate non-surgical method to treat gingival smile. By injecting it into overactive muscles, muscle activity is reduced, relaxing the lip muscles and reducing the upward pull of the lip. The improvement is almost immediate but lasts only 2 to 3 months before slowly disappearing ^13^. Little has been described evaluating repositioning, but as studies are limited, especially those focusing on the outcome, it isn't easy to define the recurrence rate ^4^. Lip repositioning improved GE by 3.4 mm (95% CI, 3.0-3.8 mm) at 6 months ^4^. A higher success rate was observed in cases where the toxin was applied before the surgical procedure, with GE by 4.5 mm at 6-month follow-up ^15^. With the assessment of muscle strength and LD verified in this study, an individualized treatment might be developed for each displacement type. This would consider different groups of LD according to strength, and based on this, the correct dose of toxin might be applied individually. Currently, the dosage of botulinum toxin is done empirically based on tactile and visual measurements. More studies are needed to indicate the dosage of botulinum toxin for each patient according to the classification proposed above.

In conclusion, the major zygomatic muscle was the most important in the smile. Individuals with LH showed a 30% increase in muscle strength compared to those with other etiologies or individuals without a gummy smile. Muscle strength increased significantly with greater lip displacement, with significant differences observed from approximately 8 mm of LD. LH, isolated or combined, was the most prevalent etiology of a gingival smile.

## Data Availability

The research data are available upon request.
